# Study on bioremediation of Lead by exopolysaccharide producing metallophilic bacterium isolated from extreme habitat

**DOI:** 10.1016/j.btre.2017.11.003

**Published:** 2017-11-08

**Authors:** Debajit Kalita, S.R. Joshi

**Affiliations:** Microbiology Laboratory, Department of Biotechnology and Bioinformatics, North-Eastern Hill University, Shillong 793022, Meghalaya, India

**Keywords:** Extremophile, *Pseudomonas*, Lead bioremediation, Epifluorescence microscopy, ICP-MS, FTIR

## Abstract

Lead released from manufacturing factories, recycling plants, automobile company and landfill leachate is abundantly found in wastewater. An efficient bioremediating agent for lead removal from wastewater is expected to ease the ever increasing problem. The present study reports *Pseudomonas* sp. W6 isolated from extreme habitat of hot water spring of North–East India evaluated for its Lead biosorption property. The bacterium showed capacity to resist 1.0 mM lead in both solid and liquid minimal media. Epifluorescence microscopy reveal the viability of bacterial cells under metal stress condition. ICP-MS analysis revealed 65% and 61.2% removal of lead from the Synthetic Bangladesh Ground Water medium in batch culture and column study respectively which was higher when compared to biosorption capacity of *P. aeruginosa MTCC*2474, *P. alcaligenes* MJ7 from forest soil and *P. ficuserectae* PKRS11 from uranium rich soil. Exopolysaccharide released by the isolate which influenced biosorption revealed the presence of ligands assayed using microbial hydrophobicity and FTIR. The extremophilic isolate is proposed as a choice for efficient bioremediation of lead contaminated wastewater.

## Highlights

• Extremophiles isolation and molecular characterization.
• Epifluorescence microscopy for the viability.
• Isolation and characterization of exopolysaccharide.
• ICP-MS analysis of Lead remediation.
• Industrially applicable testing for wastewater treatment.

## 1 Introduction

Lead (Pb) has been a potential hazardous pollutant. Industrially, Pb is used for the production of nuclear reactors protection shield, thin sheets of electronic components, Tetra-ethyl lead {(CH_3_CH_2_)_4_Pb} for vehicles, battery plates, paints, ceramics, cables and ammunition [1], [2], [3]. Acute, symptomatic Pb poisoning is most commonly detected among children in developing countries and populations living in and/or nearby Pb-polluted sites [4].

There are processes that have been developed to remediate or recover heavy metals from contaminated environments [5]. Various approaches such as physical and chemical approaches although capable of removing wide variety of contaminants carry associated disadvantages like increased energy consumption, the need of additional chemicals, pollution by byproducts etc [6]. Bioremediation on the other hand is eco-friendly process, where the pollutants can be remediated or detoxified from the soil and water using microorganisms [7].

Biological treatment of wastewater is an important environment friendly technology in which cell surface hydrophobicity/hydrophilicity (CSH) of microbial cells play a pivotal role in treatment by forming aerobic and anaerobic micro-granules [8]. Formation of biofilms and removing of contaminants from soil and water by hydrophobic and hydrophilic cells is crucial for the process (Krasowska and Sigler, 2014).

**Fig. 1:**
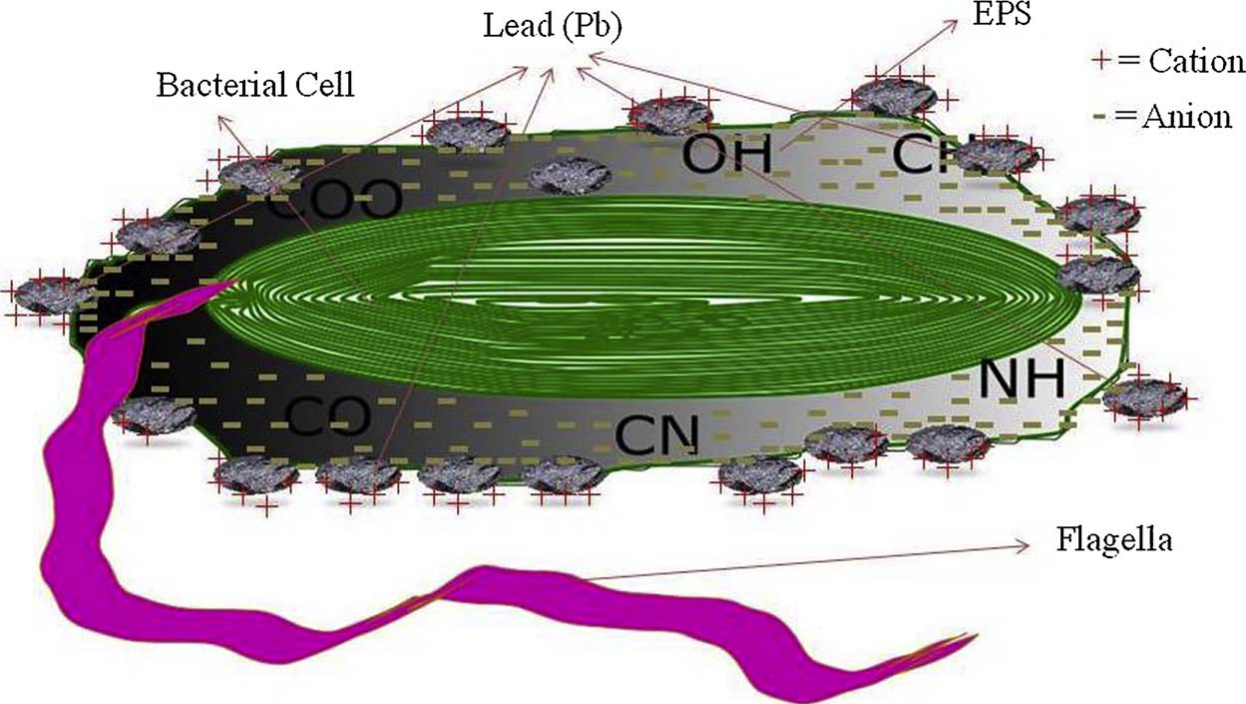
Diagrammatic representation of hydrophilic anionic EPS of bacterial cell containing carbonyl (C=O), phosphate(PO), cyanide(C=N), hydroxyl (—OH) and amino (—NH) groups that bind to cationic lead(Pb).

An effective and safer approach to metal decontamination and wastewater treatment is biofilm-mediated bioremediation (Fig. 1). Research interest in the exopolysaccharides (EPS) of microorganisms and its uses in bioremediation and bioleaching has increased due to their wide structural, physical and chemical diversity [9], [10]. The chemical composition of EPS involved in flocculation are capable of binding with metallic ions to remove heavy metals from the environment [11]. Some bacteria involved in bioremediation of toxic heavy metals include *Enterobacter* and *Pseudomonas* species [11]. Microorganisms synthesize extracellular polymers (EPs) that bind cations of toxic metals protecting metal-sensitive and essential cellular components [12].

Diverse archaea and bacteria are known to inhabit alkaline, mesophilic hot springs [13] such as *Bacillus thermoleovorans* IHI-91 [14], *Sulfolobus tengchongensis* sp. [15], *Tepidimonas taiwanensis* sp. [16], *Desulfomicrobium thermophilum* [17], *Pyrococcus* sp. [18], *Thermotoga* sp. strain PD524 [19]. Ekundayo and Killham [20] reported the solubilization and accumulation of lead by two strains of *Pseudomonas* at concentrations of 0.03 and 0.07 mg mL^−1^. Sarma et al. [21] isolated *Pseudomonas aeruginosa* DPs-13 from uranium rich subsurface soil having the capacity of biosorption of uranium upto 94% from 100 μM solution of uranyl nitrate.

Sam et al. [22] investigated the flocculation dynamics of EPS produced by a halophilic bacteria which exhibited turbidity and particle removal efficiency comparable with commercial cationic, nonionic and anionic synthetic polyelectrolytes. Cabuk et al. [23] reported that hydroxyl and carboxyl groups, as well as nitrogen based bio-ligands including amide and sulfonamide were involved in the binding of Pb(II) by *Bacillus* sp. ATS-2. [24] used microbial flocculant GA1 (MBFGA1) to remove Pb(II) ions from aqueous solution and reported removal efficiency of Pb(II) up to 99.8% by MBFGA1.

The Salt Aggregation Test (SAT) uses a salting-out agent to induce aggregation of cells for determining bacterial cell surface hydrophobicity [25]. Another commonly used technique is the Microbial Adherence to Hydrocarbons (MATH), previously called BATH (Bacterial Adherence to Hydrocarbons) [26] which can measure complicated interplay of long-range Vander Waals, electrostatic and various short-range interactions [27]. To assess the viability of microbial cells, a number of fluorescence techniques have been introduced over the past decades [28], [29]. Fluorescein and its derivatives have been used as viability probes for a wide range of microorganisms [30].

The novelty of the present work is the exploration of *Pseudomonas* sp. isolated from a extremophilic habitat of hot water spring in North-Eastern India for biosorption of Pb from wastewater. This is the first report on the biosorption capacity of a metallophilic extremophile *Pseudomonas* sp. isolated from the hot spring to evaluate bioremediation potential of heavy metals.

## 2 Materials and methods

### 2.1 Site selection and samples collection

Water samples were collected from the extreme habitat of a Hot Water Spring (vernacular: Garampani meaning hot water) in Karbi-Anglong district of Assam, India located within the Garampani Wildlife Sanctuary (26°25′12″ N, 93°43′30″ E; Survey of India Toposheet 83F/6,10 & 11 and Field Season Project (FSP) identity is 2014541 [31].

Samples were collected by using 5 point square methods [32] and precaution measures were taken according to SESDPROC-011-R4 [33] protocol. Serial dilutions of samples were made with 0.85% saline water and plated in triplicate on Plate Count Agar Media (Himedia, India) for enumeration of total viable bacteria. Inoculated plates were kept in incubators at 40 °C for 24 h [34].

### 2.2 Screening of resistant isolate

Analytical grade of Lead nitrate (PbNO_3_) (Himedia, India), was used to prepare stock solutions (100 mM/ml) of the metal. The solution was filter sterilized through a 0.2 μ nitrocellulose membrane filter disc (Millipore, India). The isolates were grown upto OD_600_ = 0.4 after 12 h in Luria Bertani (LB) broth media (Himedia, India). The cells were then re-inoculated into the Low Phosphate Medium (Tris-14.5gm/L, NaCl- 4.68gm/L, KCl-1.5gm/L, NH_4_Cl-1.0gm/L, Glycerol–5 ml/L, Na_2_SO_4_-0.043gm/L, CaCl_2_-0.03gm/L and pH-7.5 by HCl) upto OD_600_ = 0.4 to acquire McFarland scale for further experiments with equal number of cells. The cells were then washed twice with 0.9% NaCl. 10 μl of the cell suspension of each isolate was spotted onto LPM plates (150 mm diameter petriplate) impregnated with concentration of 0.5 mM, 1.0 mM and 1.5 mM of Lead nitrate (PbNO_3_) [35] and incubated at 37^ °^C for 24 h. Media without metals was considered as control. Phenazine pigment-producing *Pseudomonas aeruginosa* MTCC2474 obtained from the Microbial Type Culture Collection and Gene Bank, Chandigarh, India, *P. alcaligenes* MJ7 isolated from forest soil (unpublished data) and *P. ficuserectae* PKRS11 isolated form uranium rich sub-surface soil [36] were considered as reference strains.

The back inoculation confirmation which is a preliminary test to check whether the cells spotted into metal impregnated plate were dead or alive was carried out for viability test. For that, the whole agar along with the spot of bacterial cells from the plate were cut by sterile surgical blade and reinoculated into 5 ml of nutrient broth (Himedia, India) medium and kept for 24 h at 37^ °^C in shaker incubator at constant speed of 120 rpm. After 24 h, the visual indication and OD_600_ was taken through spectrophotometer to confirm the viability. 50 μl of the culture was added to Luria Bertani (LB) (Himedia, India) plate to count the colonies.

### 2.3 Epifluorescence microscopy

This experiment was done by using Leica SFL4000 (Leica Microsystems CMS GmbH, Germany) microscope. 12hr culture of OD_600_ = 0.4 was centrifuged and pallet was washed three times with PBS buffer (pH = 7.0). 1:5 μl of Cy3 and Cy5 dye was added to the tube and kept in dark for 30 min. 3 μl of each treated and untreated bacterial sample was taken in grease free slide and visualized under the microscope. Bacterial cells without metal was taken as control.

### 2.4 Identification of bacterium

The isolates were subjected to gram staining, biochemical (catalase, oxidase, indol, MR-VP) and sugar utilization (lactose, xylose, maltose, fructose, dextrose, galactose, raffinose, trehalose, melibiose, sucrose, L − arabinose, mannose, inulin, sodium gluconate, glycerol, salicin, dulcitol, inositol, sorbitol, mannitol, adonitol, arabitol, erythritol, α methyl D glucoside, rhamnose, cellobiose, melezitose, α methyl-D- mannoside, xylitol, ONPG, esculin hydrolysis, D arabinose, citrate utilization, malonate utilization, sorbose) tests (HiCarbo Kit KB009- Part A, Part B, Part C—Himedia, India). A single isolated colony was inoculated in 5 ml of Brain Heart Infusion Broth (Himedia, India) and incubated at 35–37 °C for 4–6 h until the inoculum turbidity reached 0.5 OD at 620 nm.

The 16S rRNA gene sequences were amplified from the genomic DNA using universal bacterial 16S rRNA primers following the protocol as described by Kumar et al. [37]. Phylogenetic neighbors were obtained using the Basic Local Alignment Search Tool (BLAST)14 program against the database of type strains with validly published prokaryotic names (available online: http://www.eztaxon.org/15) for each of the selected isolates. Molecular Evolutionary Genetics Analysis software (MEGA version 6) was used for phylogenetic analyses.

### 2.5 Bioremediation and leaching experiment

Lead nitrate(PbNO_3_)(Himedia, India) concentration was measured according to standard procedure of US Environmental Protection Agency (EPA) [38]. One mL of 12 h old bacterial culture containing 0.5 McFarland Scale (∼1.5 × 10^8^ cell/mL) was used to inoculate 50 ml SBGW (4.0mM-Na_2_HAsO_4_·7H_2_O; 0.14 M- Na_2_HPO_4_·7H_2_O; 0.45 M − NaHCO_3_, 0.84 mM-CaSO_4_·2H_2_O, 0.032M-MgCl_2_·6H_2_O, and 0.14 M-CaCl_2_) [39] containing 1 mM/L PbNO_3_, and the same medium without inoculation was used as a control. The culture were incubated at room temperature for 12 h and then centrifuged at 4000 rpm for 30 min. Supernatant was then placed into a porcelain container and acid digested. The whole sample was analyzed using ICP-MS for lead detection. The percentage of metal removal/adsorption was calculated as:

(1) $$\%\;Rem.\;(Pb)=\frac{C_{Initial}-C_{Final}}{C_{Initial}}\times 100$$

where, *C*_initial_ is the initial metal concentration and *C_Final_* is the metal concentration after contact with the bacterial culture (mg L^−1^) [40].

Leaching experiments were conducted by following the method of Klute and Dirksen [41] with minor modifications by placing 33 g of soil impregnated with bacterial biomass in 48 cm × 2 cm glass columns. The height of soil bed volume was 17.28 cm. Soil without bacterial biomass was taken as control. Columns were initially wetted to saturation from the bottom to avoid entrapment of air in the soil pore space. Solution was poured above the soil layer in column to a depth of 2.0 cm for equilibration. After the equilibration period, a peristaltic pump with a constant flow rate of 100 μl/min was employed to pump the SBGW in the column with an effluent tube fixed to a fraction collector of 10 ml. Leachate solutions were collected in increments of approximately 50 ml initially and increasing to approximately 100 ml at the end of leaching. The pH and calcium ion activity of each effluent sample was measured immediately after collection. The concentration of Pb in each extract solution was also measured by atomic absorption spectroscopy. After leaching, the soil was removed from each column with a plunger and divided into three sections, each of 10 gm. The final soil pH was measured as a saturated paste. Total residual Pb concentration in the leached soil was measured following digestion in aqua regia and hydrofluoric acid [42].

### 2.6 Microbial adherence tests

Salt aggregation test (SAT): Twofold serial dilutions of ammonium sulfate [(NH_4_)_2_SO_4_] (SRL, India) in PBS pH 6.8, ranging from 0.007 M to 4 M, were prepared [25]. 50 μl aliquots of each ammonium sulfate solution were placed on glass slides and mixed thoroughly with 50 μl of bacterial suspensions (∼10^11^ CFU) resuspended in PBS. The lowest amount of ammonium sulfate giving visible bacterial clumping was scored as a numerical value of the level of bacterial surface hydrophobicity (SAT value). The SAT values of <2 M for the isolates were considered as positive [43].

Polystyrene adherence test (PAT): Aliquots of 50 μl of bacterial suspension (∼10^11^ CFU) in PBS, pH 6.8 were poured on the surface of polystyrene Petri dishes (Falcon, USA). The plastic plates were then vertically positioned to allow the droplets to drain. The plastic surface was washed with distilled water and fixed with methanol (SRL, India). During this procedure, non-adherent microorganisms were washed off. Bacterial cells that remained bound to the polystyrene plate were stained with 1% crystal violet solution (Himedia, India) [43].

Microbial adherence to *n*-hexadecane test (MATH): Aliquots of 4 ml of bacterial cell suspension (∼3 × 10^8^ CFU) in PBS, pH 7.2 were overlaid with 400 μl of *n*-hexadecane (SRL, India), and incubated in a water bath at 37 °C for 10 min. The suspensions were vortexed. Phase separation was obtained after 15–20 min at room temperature. The percentage of partitioning in the hydrocarbon phase was calculated as follows: {OD_640nm_ (bacterial suspension) − OD_640nm_ (aqueous phase)}/OD_640nm_ (original bacterial suspension) X 100. Strains considered highly hydrophobic gave values ≥ 50%, and those moderately hydrophobic ranged from >20 to <50%. Hydrophilic surfaces have values ≤ 20% [26], [43].

### 2.7 Extraction and characterization of exopolysaccharide

Bacterium were subcultured and slants were inoculated and maintained at 37 °C for 24 h. Experiments were done using 250 ml flask each containing 100 ml of basal medium (Dextrose −10 gm; yeast extract − 3 gm; malt extract − 3 gm; peptone − 5 gm; MgSO_4_. 7H_2_O − 1 gm; KH_2_PO_4_–0.3 gm) and Vitamin B_1_(10 mg) incorporated at 37 °C on an orbital shaker incubator at 150 rpm for 72 h.

After 72 h of incubation, basal medium was centrifuged at 5000 rpm for 20 min. The EPS was then precipitated from the supernatant by addition of equal amount of ethanol. The mixture was agitated with addition of methanol to prevent local high concentration of the precipitate and left overnight at 4 °C and centrifuged at 7000 rpm for 20 min. After centrifugation, the precipitate was collected in a Petri plate and dried at 60 °C [44].

The purified bioflocculant (2 mg) was ground with 100 mg KBr and compressed at 7500 kg for 3 min to obtain translucent pellets. KBr pellet was used as the background reference. Infrared absorption spectra were verified with FTIR (Perkin Elmer FTIR-400, USA). The spectral resolution and wave number accuracy were 4 and 0.01 cm^−1^ respectively.

All experiments were conducted in triplicates (n = 3) and results were analyzed and reported as ±SE. Student T-test was done to check the statistical significance of the findings. OrginPro8 was used for all analysis.

## 3 Results

### 3.1 Description of site, isolation, screening of metal tolerance ability, epifluorescence microscopy and characterization

**Fig. 2:**
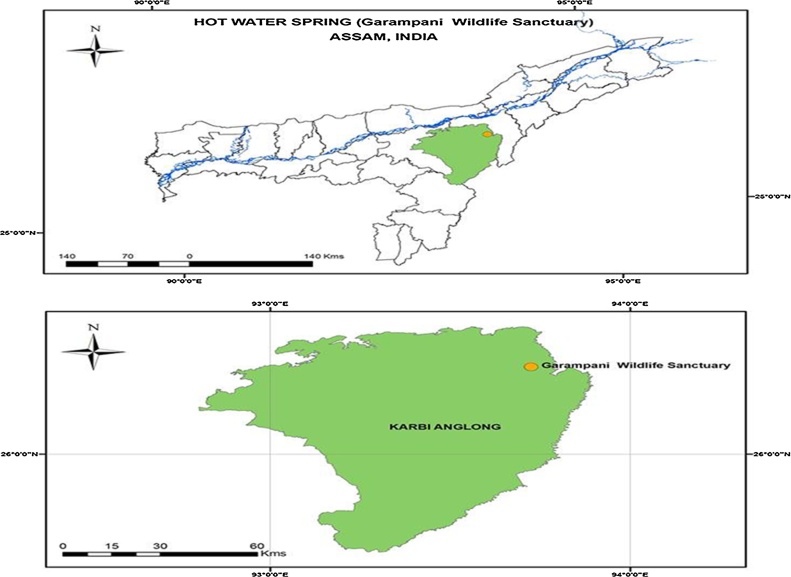
GPS mapping of studied Hot Water Spring. The yellow point indicates the sampling site located in Garampani wildlife sanctuary.

**Fig. 3:**
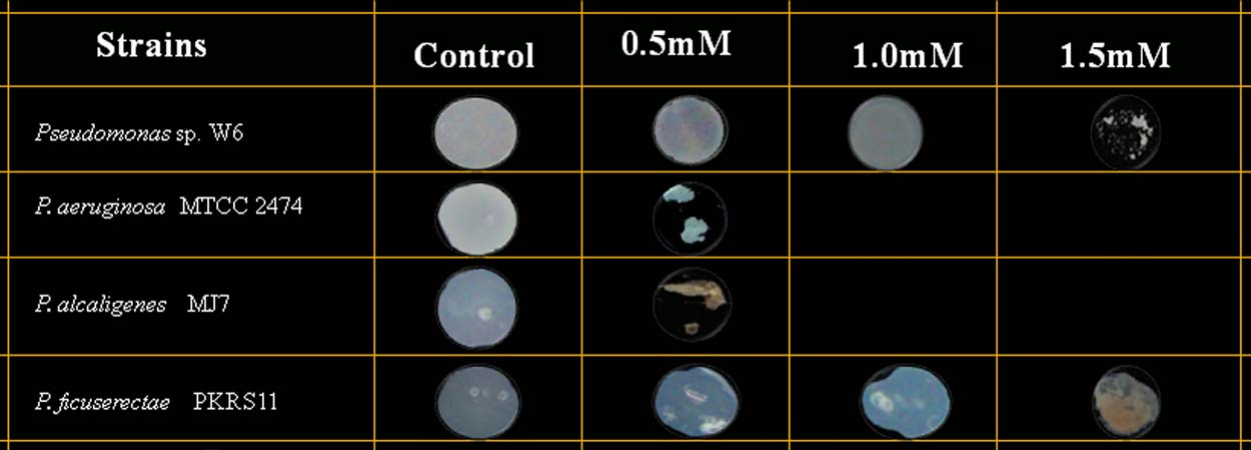
Lead tolerance by *Pseudomonas* sp. W6, *Pseudomonas aeruginosa* 2474 (MTCC), *Pseudomonas alcaligenes MJ7* and *Pseudomonas ficuserectae* PKRS11. The picture represents the bacterial growth under control, 0.5 mM and 1.0 mM and 1.5 mM Pb concentration.

**Fig. 4:**
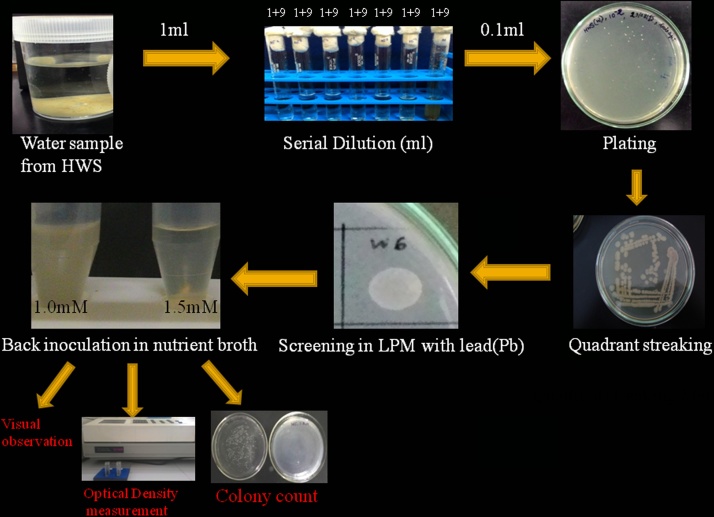
Schematic representation of work flow used in the study.

**Fig. 5:**
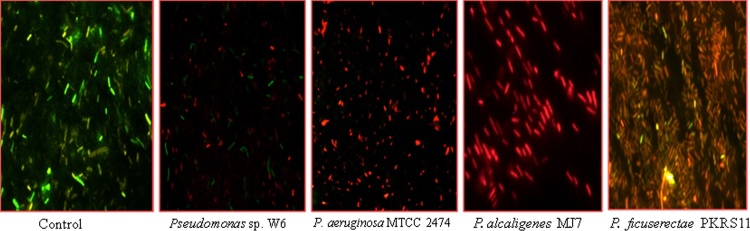
Epifluorescence microscopy of Live and Dead cells in control and MIC of *Pseudomonas* sp. W6, *Pseudomonas aeruginosa* 2474 (MTCC), *Pseudomonas alcaligenes MJ7* and *Pseudomonas ficuserectae* PKRS11.

**Fig. 6:**
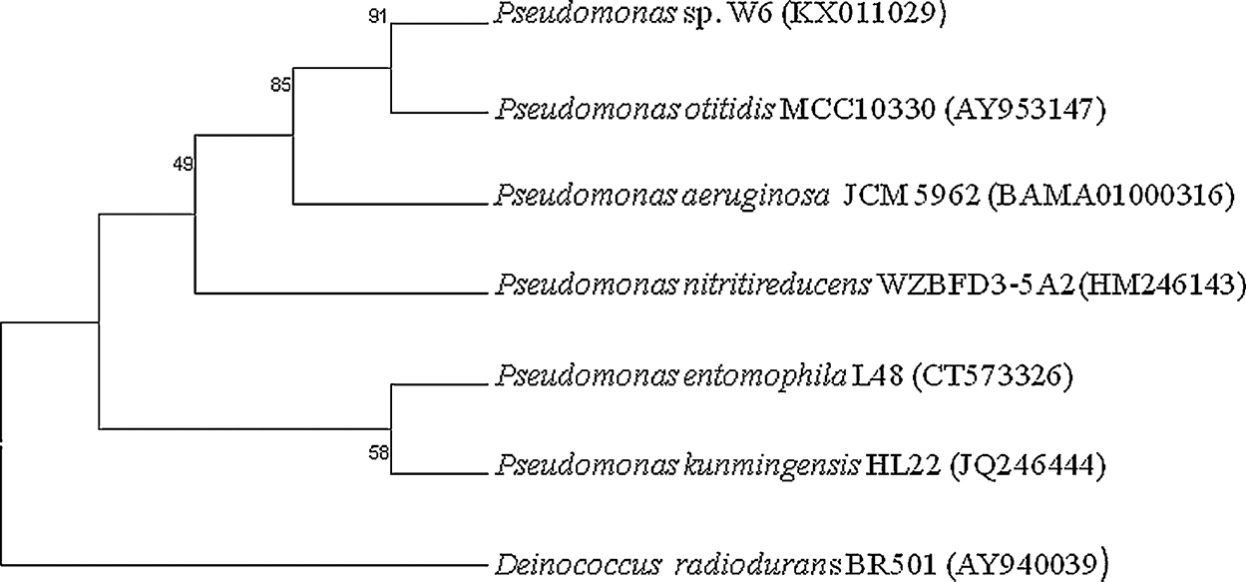
Phylogenetic tree of *Pseudomonas* sp. W6 constructed using Neighbor Joining method.

Hot water spring site was selected being an extreme environment and proper location of sample collected site and its GPS mapping was done by Arc GIS 9.3 software by the university experts in collaboration with Space Application Centre, ISRO (Fig. 2). The physico-chemical parameter of the site is given in Table 1. Out of the five bacterial isolates, w6 showed tolerance upto 1 mM of Pb (Fig. 3). The back inoculation was done to confirm the viability of cells (Fig. 4). Culture in 1.0 mM Pb had extensive growth of bacterium, whereas 1.5 mM inhibited bacterial growth which was confirmed by spectrophotometric method and plate count. In an earlier report of Rakshak et al. (2013), *Pseudomonas ficuserectae* PKRS11 was able to tolerate upto 1.0 mM of Lead. The other two type strains *P. aeruginosa* MTCC2474 and *P. alcaligenes* MJ7 were found to tolerate up to 0.5 mM Pb. The investigation of all bacterial viability under metal stressed condition was also studied by epifluorescence microscopy. Cy3 absorbs the wavelength in 550 nm and releases at 570 nm while Cy5 absorbs the wavelength in 650 nm and releases it at 670 nm, which is detected by fluorescence microscope with adjustable filter. This fluorescing property allows the detection of dead cells clearly differentiated from the live cells. Dead cells appear red in colour while live cells fluoresce the green colour (Fig. 5). Strain W6 could grow at various temperatures ranging from 37 to 45 °C with optimal growth at 40 °C and pH 7 and revealed a wide range of sugar utilization capacity (Table 2). The bacterium was identified upto the genus level as *Pseudomonas* sp. using Bergey’s Manual of Determinative Bacteriology [45]. The 16S rRNA sequencing was used for generating the phylogeny and sequences submitted to Genbank with accession number KX011029. The phylogenetic tree was constructed on the basis of neighbor joining method making a single clade match with *Pseudomonas* sp.(KT375336) with *Deinococcus radiodurans* as a outgroup (Fig. 6).

**Table 1:** Physical parameters observed for the sampled Hot Water Spring.

Parameters	Data
Temperature	38 ± 2 °C Winter, 46 ± 2 °C Summer
pH	8.4 ± 2
Humidity	92.2 ± 2%

**Table 2:** Sugar utilization by *Pseudomonas* sp. W6 observed using HiCarbo Kit KB009 (+ve indicates sugar utilization and −ve indicates lack of sugar utilization capacity of the isolate).

Sl.No.	Sugars	Results
1	Lactose	-Ve
2	Xylose	-Ve
3	Maltose	-Ve
4	Fructose	-Ve
5	Dextrose	-Ve
6	Galactose	-Ve
7	Raffinose	-Ve
8	Trehalose	-Ve
9	Melibiose	-Ve
10	Sucrose	-Ve
11	L − Arabinose	-Ve
22	Mannose	-Ve
13	Inulin	-Ve
14	Sodium gluconate	-Ve
15	Glycerol	-Ve
16	Salicin	-Ve
17	Dulcitol	-Ve
18	Inositol	-Ve
19	Sorbitol	-Ve
20	Mannitol	+Ve
21	Adonitol	-Ve
22	Arabitol	-Ve
23	Erythritol	-Ve
24	α methyl D glucoside	-Ve
25	Rhamnose	-Ve
26	Cellobiose	-Ve
27	Melezitose	-Ve
28	α methyl-D- mannoside	-Ve
29	Xylitol	-Ve
30	ONPG	-Ve
31	Esculin hydrolysis	-Ve
32	D Arabinose	-Ve
33	Citrate Utilization	+Ve
34	Malonate Utilization	+Ve
35	Sorbose	-Ve
36	Control	Pink

### 3.2 Cell surface characterization, adherence profiling, Pb remediation of *Pseudomonas* sp. of synthetic Bangladesh ground water (SBGW)

**Fig. 7:**
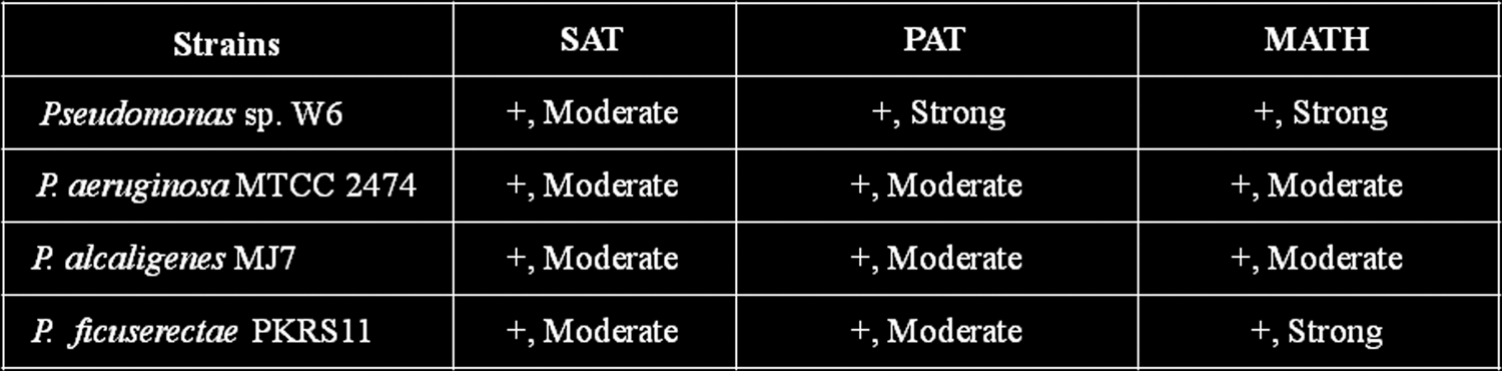
SAT, PAT and MATH test of *Pseudomonas* sp. W6, *Pseudomonas aeruginosa* 2474 (MTCC), *Pseudomonas alcaligenes MJ7* and *Pseudomonas ficuserectae* PKRS11.

**Fig. 8:**
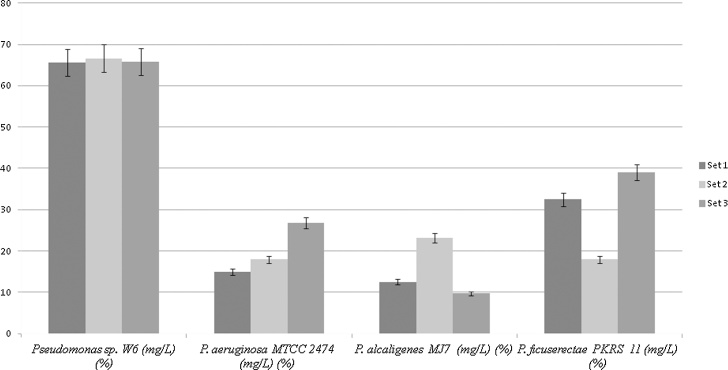
ICP-MS data for control and Pb-treated samples. The graph depicts the two line, where upper line represents the amount of Pb present in control mg/L and lower line represents the amount of Pb present in medium broth after 12 h treatment (Observations are presented in mg/L for 3 sets of data).

The criteria used to detect cell surface hydrophobicity are tabulated in Table 3. The degree of hydrophobicity for SAT is positive and moderate while for PAT and MATH are positive and strong respectively. The hydrophobicity test indicated *Pseudomonas* sp.W6 having capacity to bind Pb in comparison to other type strains. *P. Aeruginosa* MTCC2474, *P. alcaligenes* MJ7 and *P. ficuserectae* PKRS11 showed positive and moderate in SAT and PAT test, but *P. ficuserectae* PKRS11 exhibited positive and strong in MATH test (Fig. 7). ICP-MS analysis indicated that concentration of Pb decreased in supernatant after 12 h in comparison to the control (Fig. 8). The bacterium *Pseudomonas* sp W6 adsorbed approximately 66% Pb from the synthetic Bangladesh ground water at optimum pH and temperature in comparison to *P. Aeruginosa* MTCC 2474 (19.9%), *P. alcaligenes* MJ7 (45.3%), *P. ficuserectae* PKRS 11 (29.8%). Lead removal capacity of *Pseudomonas* sp. W6 in synthetic water revealed Pb remediation in natural water as well. The soil column experiment showed lead removal from leachate as 4.71, 46.66, 50.07, 19.21 and in soil as 60.29, 54.32, 42.77, 57.45 by *P. aeruginosa* W6, *P. aeruginosa* MTCC 2474, *P. alcaligenes* MJ7 and *P. ficuserectae* PKRS 11 respectively (Fig. 10). The results of metal analysis of the soil column used in the leaching process indicated bacterial embedded soil having more bioremediation efficiency when compared to soil without bacteria. Soil pH before and after leaching was found to be 7.21 and 5.67 respectively as Pb becomes acidic when it dissolves in water thus reducing the pH. Soil macropores were observed to be filled with gas bubbles beginning at the top of the columns, especially in columns A and B. The gas bubbles are presumably CO_2_ caused by the dissolution of calcium carbonate. The presence of gas in the macropores caused a change from saturated to unsaturated flow.

**Table 3:** Criteria to analysis the surface hydrophobicity.

Test	Value	Degree of hydrophobicity	Result
SAT	0.0 < 1.0 M	Strong	Positive test
	1.0 < 2.0 M	Moderate	Positive test
	2.0 < 4.0 M	Weak	Negative test
	≥ 4.0 M	Not Hydrophobic	Negative test
PAT	Thick complete layer	Strong	Positive test
	Thin complete layer	Moderate	Positive test
	No bound cells	Not hydrophobic	Negative test
MATH	≥50%	Strong	Positive test
	˃20–<50%	Moderate	Positive test
	≤ 20%	Not hydrophobic	Negative test

### 3.3 Extraction and metal binding ligand characterization of metallophilic exopolysaccharide

**Fig. 9:**
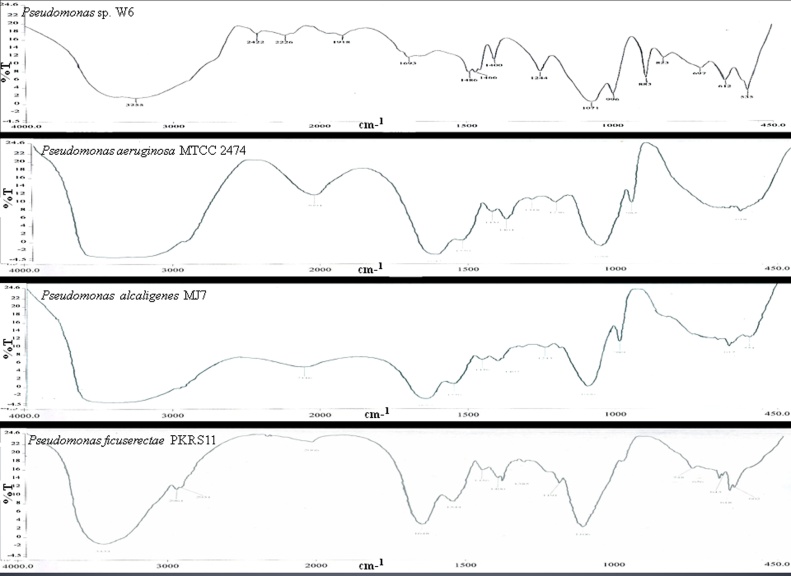
FTIR of EPS showing wavelength of *Pseudomonas* sp. W6, *Pseudomonas* sp. W6, *Pseudomonas ficuserectae* PKRS 11, *Pseudomonas aeruginosa* MTCC 2474 and *Pseudomonas alcaligenes* MJ7. The band present in the EPS correspond to the groups having property to bind with the metals.

Exopolysaccharide was extracted from the bacterium and lyophilized. The Infrared spectra of the bacterial EPS depicted the functional groups that are responsible for binding the heavy metal ion with bacterial cell surface (Fig. 9). Infrared absorption bands and their corresponding groups with intensity of EPS of *Pseudomonas* sp. W6 exhibited the wave numbers with the corresponding groups. The band of 3255 cm^−1^, 2422 cm^−1^, 1486 cm^−1^ corresponded to —OH and −NH groups, C=O and —COO groups, −C—O and C—N groups respectively with strong and medium intensity. The band of 2226,1466,1400,1244, 883,823, 697 and 612 corresponded to C—H bonds with strong and medium intensity. The functional groups of wave numbers 1918 and 996 are C—C with strong and medium intensity and 1071 is C—O with medium or strong intensity. The FT-IR analysis showed the coordination of metals with functional groups present in the EPS. The amino, cyanide, hydroxyl, carbonyl and carboxyl ligands provide the major biosorption sites for metal binding. *P. aeruginosa* MTCC2474 showed band 3440 cm^−1^, 2094 cm^−1^, 1647 cm^−1^, 1550 cm^−1^, 1451 cm^−1^, 1404 cm^−1^, 1088 cm^−1^, 985 cm^−1^ corresponding to —OH and −NH groups, C=O and —COO groups, −C—O and C—N, −CH, C—C, C—N,-C—O and C—N, C—H and C—O groups with strong and medium intensity, whereas 1318 and1236 has medium intensity showing the −CH match. *P. alcaligenes* MJ7 represented 3429 cm^−1^, 2116 cm^−1^, 1550 cm^−1^, 1456 cm^−1^, 1402 cm^−1^, 989 cm^−1^ and 617 cm^−1^ resembling with —OH and −NH groups, C—N, C—C,-C—O and C—N, C—H and C—O, which provides strong intensity.1650 cm^−1^, 1243 cm^−1^ and 1096 cm^−1^ conform the −CH of medium intensity. *P. ficuserectae* PKRS 11 corresponded to 3434 cm^−1^,1985 cm^−1^ and 1191 cm^−1^ exhibiting strong intensity with functional groups of —OH and —NH, C—H and C—O. Maximum functional groups match with weak and medium intensity bands such as 2961 cm^−1^, 2931 cm^−1^,2066 cm^−1^,1648 cm^−1^ and 748 cm^−1^ as weak and 1544 cm^−1^,1456 cm^−1^,1400 cm^−1^,1106 cm^−1^, 656 cm^−1^ and 643 cm^−1^ showed medium intensity in metal binding (Table 4).

**Fig. 10:**
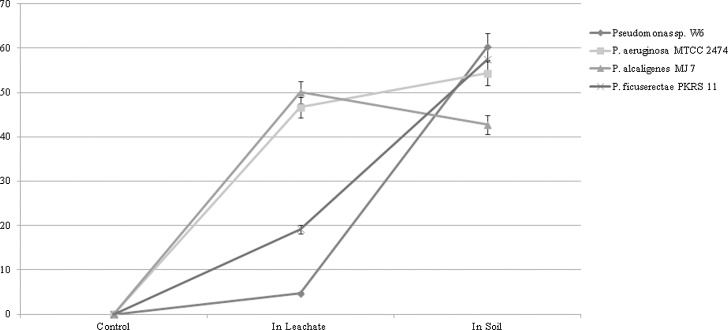
Percentage of Pb remaining in leachate and soil after column leaching experiments of *Pseudomonas* sp. W6, *Pseudomonas aeruginosa* MTCC 2474, *Pseudomonas alcaligenes* MJ7 and *Pseudomonas ficuserectae* PKRS11.

**Table 4:** FTIR characterization of EPS showing wavenumbers (cm^−1)^, functional groups and intensity of *Pseudomonas* sp. W6, *Pseudomonas ficuserectae* PKRS 11, *Pseudomonas aeruginosa* MTCC 2474*, Pseudomonas alcaligenes* MJ7.

Wave numbers, cm^−1^ Pseudomonas sp. W6	Functional groups	Intensity	Wave numbers, cm^−1^ (*P. ficuserectae* PKRS 11)	Functional groups	Intensity	Wave numbers, cm-^1^ (*P. aeruginosa* MTCC *2474*)	Functional groups	Intensity	Wave numbers, cm^−1^ (*P. alcaligenes MJ7*)	Functional groups	Intensity
3255	—OH, —NH	Strong, Medium	3434	-OH, −NH	Strong, Medium	3440	-OH, −NH	Strong, Medium	3429	-OH, —NH	Strong, Medium
2422	C=O, —COO	Strong	2961	C—H	Weak	2094	C=O, —COO	Strong	2116	C—N	Strong
2226	-CH	Strong	2931	C—H	Weak	1647	—CH	Strong	1650	—CH	Medium
1918	C—C	Strong, Variable	2066	C—H	Weak	1550	C—C	Strong, Variable	1550	C—C	Strong, Variable
1693	C—N	Strong	1648	C—H	Weak	1451	C—N	Strong	1456	C—N	Strong
1486	—C—O,C—N	Strong	1544	C=C	Medium	1404	—C—O, C—N	Strong	1402	-CO, C—N	Strong
1466	—CH	Strong	1456	-CH	Medium	1318	-CH	Medium	1243	—CH	Medium
1400	—CH	Medium	1400	-CH	Medium	1236	-CH	Medium	1096	—CH	Medium
1244	C—H	Strong	1385	C—H	Strong	1088	C—H	Strong	989	C—H	Strong
1071	C—O	Medium/ Strong	1191	C—O	Medium/ Strong	985	C—O	Medium/ Strong	617	C—O	Medium/ Strong
996	C—C	Medium	1106	C—C	Medium						
883	C—H	Weak	748	C—H	Weak						
823	C—H	Medium	656	C—H	Medium						
697	C—H	Medium	643	C—H	Medium						
612	C—H	Medium									

### 3.4 Analysis of significant data statistically

Statistical analysis showed that the two tailed P value of less than 0.0001 and this difference is considered to be statistically significant at 95% confidence interval with standard error of 0.007.

## 4 Discussion

*Pseudomonas* is found in all environment due to its tolerance, but limited reports are available on metallophilic *Pseudomonas* species from hot water sparing especially from North-East India, a biodiversity hot-spot of the world and this is first time report on the metallophilic exopolysaccharide producing bacterium having properties of Pb bioremediation. *Pseudomonas* sp. designated as W6 was isolated from the hot water spring by using serial dilution method. The present work describes Lead biosorption property of *Pseudomonas* sp. W6 isolated from extreme habitat of hot water spring of North–East India. The bacterium revealed the tolerance capacity to resist 1.0 mM lead in both solid and liquid minimal media with bioremediation potential.

The application of metallophilic *Pseudomonas* is a new approach to remove metals from ground water. Al-Aoukaty et al. [46] investigated that *P. fluorescens* ATCC13525 accumulated lead extra-and intra-cellularly in presence of phosphate. An alkaliphilic bacterium, *Pseudomonas pseudoalcaligenes* CECT5344 is appropriate organisms to treat wastewaters containing cyanide where it uses cyanide as nitrogen source for growth [47]. Three lead tolerant strains of *Pseudomonas* namely, B6, D4 and E4 were found to tolerate lead at MIC values over 600 mg l^−1^ [48]. All earlier reports on lead removal study by *Pseudomonas* are on nutrient rich and/or low medium, but there are no reports of synthetic Bangladesh ground water (SBGW) except for 92.7% by *Paenibacillus polymyxa* from aqueous solutions [49], 85.4% by *Bacillus cereus* [50], 84% biosorption by *Streptomyces* VITSVK5 [51]. Cao et al. [52] reported *Pseudomonas* sp. CY63, isolated from hyphosphere contaminated soils having Pb accumulation capacity of 158.9%. The experiments of remediation in SBGW were done to observe the remediation percentage in spike water which resembles natural water. ICP-MS analysis showed 65% removal in batch culture and 61.2% removal in column study of lead from the Synthetic Bangladesh Ground Water which was higher in comparision to *P. aeruginosa* MTCC2474, *P. alcaligenes* MJ7 from forest soil and *P. ficuserectae* PKRS11 from uranium rich soil. The results of the present study reveal the bacterial viability under metal stressed condition as observed under fluorescence analysis. Epifluorescence microscopy established the cells viability under metal stress condition when compared to observations made for other organisms in the study.

In case of column tests the approach of a standardized contact time enables comparable results despite different column dimensions [53], [54]. *Bacillus subtillis* and *E. coli* were employed to study column remediation experiment of cadmium, which increased the retardation of Cd inside the column bed [55]. The experimental results showed *Pseudomonas* sp. having capacity to absorb Pb impregnated in soil as revealed by soil column analysis. Some cationic metals like copper prefer binding to neutral amino groups, whereas some, such as lead, form negatively charged complexes in water such as Pb(OH)^3−^ and Pb(OH)_4_^2−^ that can interact electrostatically with positively charged amino groups [56]. Microorganisms bearing hydrophobic/hydrophilic properties are useful in bioremediation processes such as degradation of hydrocarbons, metals or biodegradable polyesters Obuekwe et al., 2009. Bacteria acquire specific adaptive mechanisms to modify chemically and physiologically their surface to hydrophobicity due to low bioavailability of the substrates and environmental toxicity. This modification enhances and permits hydrophobic–hydrophobic interactions with the substrates [57]. Torres et al. [58] reported gram-positive bacterium, *Bacillus licheniformis* exhibiting reduction in cell surface hydrophobicity and showed affinity towards toxic organic compounds in presence of organic solvents. Cell surface hydrophobicity is measured to check the adherence with solid or liquid particles. MATH has promoted researchers in a wide variety of fields to consider the hydrophobic effect when discussing microbial adhesion. Bacterial hydrophobicity is one the many parameters which determine the ability of a cell to adhere, invade and cause damage [59]. Hydrophobic microorganisms are capable of adhering to oil/water interface and utilizing oil components as a source of energy for growth and metabolism [60]. *Pseudomonas* sp. W6 showed high adherence capacity towards the metal substrate and/or metals in soluble form as observed using SAT, PAT and MATH. Cell surface hydrophobicity ability of *Pseudomonas* sp.W6 exhibited its ability to bind the metals from the environment. Cell free extract of *Pseudomonas* sp. sorbs lead ions more effectively than whole cells from aqueous solutions [61]. Hydrophilic microbes are more resistant to organo-metallic compounds and solvent resulted by the modification of cell membrane lipopolysaccharide [62]. The fast and proper dispersion of hydrophilic strains having low affinity towards adhesion are therefore advantageous in bioremediation processes [63]. The findings demonstartes *Pseudomonas* sp. W6 bacterial adhesion to liquid hydrocarbons providing a strong evidence of the hydrophobic surface adherence properties of this bacterial isolate.

High-resolution ^31^P nuclear magnetic resonance spectroscopy revealed *Pseudomonas putida* ATCC 33015 binding germanium and lead ions to lipopolysaccharide [64]. Carbonyl(C=O), phosphate (PO), hydroxyl (—OH) and amino (-NH) groups had this role in *Pseudomonas aeruginosa* ASU6a [65], [66]. Pb(II) binding by EPs has been reported for *Bacillus firmus*, *Pseudomonas* sp. [67]. In case of *Pseudomonas marginalis* isolated from metal contaminated soil, EPs had the capacity to bind Pb(II) up to 2.5 mM (0.3 mM of soluble lead in minimal medium, pH 6) [68]. The EPS characterized using FTIR for *Pseudomonas* sp.W6 also showed the presence of carbonyl (C=O), phosphate(PO), cyanide(C=N), hydroxyl (—OH) and amino (—NH) groups with high strong binding capacity with the metals. Exopolysaccharide released by this isolate influenced biosorption as revealed the presence of ligands assayed using microbial hydrophobicity and FTIR.

## 5 Concluding remarks

An increasing concentration of hazardous heavy metals like Pb(II) in the environment specially in water has stimulated research to look for new possible ways for its removal/neutralization. In the present study, a novel metallophilic hydrophilic *Pseudomonas* sp. isolated from the hot water spring offers advantage with EPS production and this bacterium can be exploited for waste water treatment. The exopolysaccharide produced by the isolate makes it effectively adhere to lead with higher affinity to bind in its ligands. Further investigation of formulation and development involved in bioflocculation process is in progress for its bioprospection. The present finding supports the exploration of adhesive and bioremediation properties of *Pseudomonas* isolate W6 which offer a low-cost and environmentally friendly technology for treatment of domestic, industrial or mixed wastewater. It is suggested as one of the choices to ensure treatment efficiency and performance for industrial effluents contaminated with metals like lead(Pb).

## Conflict of interest

There are no conflict of interest declared by the authors.

## References

[1] Sparks D.L. (2005). Toxic metals in the environment: the role of surfaces. Elements.

[2] ATSDR (2007).

[3] UNEP (2009). http://www.unep.org/pcfv.

[4] WHO (2010).

[5] Akinci G., Guven D.E. (2011). Bioleaching of heavy metals contaminated sediment by pure and mixed cultures of *Acidithiobacillus* spp. Desalination.

[6] Ilhan S., Kilicrslan S., Odag H. (2004). Removal of chromium, lead and copper ions from industrial waste waters by *Staphylococcus saprophyticus*. Turk. Electron. J. Biotechnol..

[7] Talley J., Jaferey W., Talley L. (2005). Bioremediation of Recalcitrant Compounds.

[8] Liu X., Sheng G., Yu H. (2009). Physicochemical characteristics of microbial granules. Biotechnol. Adv..

[9] Fialho A.M., Moreira L.M., Granja A.T., Popescu A.O., Hoffmann K., Sá-Correia I. (2008). Occurrence, production, and applications of gellan: current state and perspectives. Appl. Microbiol. Biotechnol..

[10] Rehm B. (2009). Microbial Production of Biopolymers and Polymer Precursors: Applications and Perspectives.

[11] Pal A., Paul A.K. (2008). Microbial extracellular polymeric substances: central elements in heavy metal bioremediation. Indian J. Microbiol..

[12] Bruins M.R., Kapil S., Oehme F.W. (2000). Microbial resistance to metals in the environment. Ecotoxicol. Environ. Saf..

[13] Coman C., Druga B., Hegedus A., Sicora C., Dragos N. (2013). Archaeal and bacterial diversity in two hot spring microbial mats from a geothermal region in Romania. Extremophiles.

[14] Markossian S., Becker B., Märkl H., Antranikian G. (2000). Isolation and characterization of lipid-degrading *Bacillus thermoleovorans* IHI-91 from an icelandic hot spring. Extremophiles.

[15] Xiang X., Dong X., Huang L. (2003). *Sulfolobus tengchongensis* sp. nov., a novel thermo-acidophilic archaeon isolated from a hot spring in Tengchong, China. Extremophiles.

[16] Chen T.L., Chou Y.J., Chen W.M., Arun B., Young C.C. (2006). *Tepidimonas taiwanensis* sp. nov., a novel alkaline-protease-producing bacterium isolated from a hot spring. Extremophiles.

[17] Thevenieau F., Fardeau M.L., Ollivier B., Joulian C., Baena S. (2006). *Desulfomicrobium thermophilum* sp. nov.: a novel thermophilic sulphate-reducing bacterium isolated from a terrestrial hot spring in Colombia. Extremophiles.

[18] Kecha M., Benallaoua S., Touzel J.P., Bonaly R., Duchiron F. (2007). Biochemical and phylogenetic characterization of a novel terrestrial hyperthermophilic archaeon pertaining to the genus *Pyrococcus* from an Algerian hydrothermal hot spring. Extremophiles.

[19] Kanoksilapatham W., Keawram P., Gonzalez J.M., Robb F.T. (2015). Isolation, characterization, and survival strategies of *Thermotoga* sp. strain PD524: a hyperthermophile from a hot spring in Northern Thailand. Extremophiles.

[20] Ekundayo E.O., Killham K. (2001). Lead solubilization and accumulation by two strains of *Pseudomonas* obtained from a contaminated Alfisol’s effluent in south western Nigeria. Environ. Monit. Assess..

[21] Sarma B., Acharya C., Joshi S.R. (2012). Plant growth promoting and metal bioadsorption activity of metal tolerant *Pseudomonas aeruginosa* isolate characterized from uranium ore deposit. Proc. Natl. Acad. Sci. India Sect. B Biol. Sci..

[22] Sam S., Kucukasik F., Yenigun O., Nicolaus B., Oner E.T., Yukselen M.A. (2011). Flocculating performances of exopolysaccharides produced by a halophilic bacterial strain cultivated on agro-industrial waste. Bioresour. Technol..

[23] Cabuk A., Akar T., Tunali S., Tabak O. (2006). Biosorption characteristics of *Bacillus* sp. ATS-2 immobilized in silica gel for removal of Pb(II). J. Hazard. Mater..

[24] Feng J., Yang Z., Zeng G., Huang J., Xu H., Zhang Y., Wei S., Wang L. (2013). The adsorption behavior and mechanism investigation of Pb(II) removal by flocculation using microbial flocculant GA1. Bioresour. Technol..

[25] Ljungh A., Hjertén S., Wadström T. (1985). High surface hydrophobicity of auto aggregating *Staphylococcus aureus* strains isolated from human infections studied with the salt aggregation test. Infect. Immun..

[26] Rosenberg M., Gutnick D., Rosenberg E. (1980). Adherence of bacteria to hydrocarbons: a simple method for measuring cell-surface hydrophobicity. FEMS Microbiol. Lett..

[27] Van der Mei H.C., van de Belt-Gritter B., Busscher H.J. (1995). Implications of microbial adhesion to hydrocarbons for evaluating cell surface hydrophobicity. 2. Adhesion mechanisms. Colloids Surf. B: Biointerfaces.

[28] Manafi M., Kneifel W., Bascomb S. (1991). Fluorogenic and chromogenic substrates used in bacterial diagnostics. Microbiol. Rev..

[29] McFeters G.A., Yu F.P., Pyle B.H., Stewart P.S. (1995). Physiological assessment of bacteria using fluorochromes. J. Microbiol. Methods.

[30] Porter J., Deere D., Pickup R., Edwards C. (1996). Fluorescent probes and flow cytometry: new insights into environmental bacteriology. Cytometry.

[31] Kumar A., Kumar R.T. (2015). http://www.portal.gsi.gov.in/pls/gsipub/PKG_PTL_SEARCH_PAGES.pViewReportDtl?inpReportId=2014541.

[32] Japan Environment Agency (1998).

[33] U.S. Environmental Protection Agency Science and Ecosystem support Division(SESDPROC) (2013).

[34] Downey A.S., DaSilva S.M., Olson N.D., Filliben J.J., Morrow J.B. (2012). Impact of processing method on recovery of bacteria from wipes used in biological surface sampling. Appl. Environ. Microbiol..

[35] Lim C.K., Cooksey D.A. (1993). Characterization of chromosomal homologs of the plasmid-borne copper resistance operon of *Pseudomonas syringae*. J. Bacteriol..

[36] Kumar R., Nongkhlaw M., Acharya C., Joshi S.R. (2013). Growth media composition and heavy metal tolerance behavior of bacteria characterized from the sub-surface soil of uranium rich ore bearing site of Domiasiat in Meghalay. Indian J. Biotechnol..

[37] Kumar R., Acharya C., Joshi S.R. (2011). Isolation and analyses of uranium tolerant *Serratia marcescens* strains and their utilization of aerobic uranium U(VI) bioadsorption. J. Microbiol..

[38] EPA (2007).

[39] BGS (2000). http://www.bgs.ac.uk/arsenic.

[40] Erdem E., Karapinar N., Donat R. (2004). The removal of heavy metal cations by natural zeolites. J. Colloids Interface Sci..

[41] Klute A., Dirksen C., Klute A. (1986). Methods of Soil Analysis Part I, Physical and Mineralogical Methods.

[42] Lim C.H., Jackson M.L., Page A.L. (1982). Methods of Soil Analysis Part II, Chemical and Microbiological Properties.

[43] Mattos-Guaraldi A.L., Formiga L.C.D., Andrade A.F.B.v. (1999). Cell surface hydrophobicity of sucrose fermenting and non fermenting *Corynebacterium diphtheriae* strains evaluated by different methods. Curr. Microbiol..

[44] Wang Y., Ahmed Z., Feng W., Li C., Song S. (2008). Physicochemical properties of exopolysaccharide produced by *Lactobacillus kefiranofaciens* ZW3 isolated from Tibet kefir. Int. J. Biol. Macromol..

[45] Holt J.G., Krieg N.R., Sneath P.H.A., Staley J.T., Williams S.T. (1994).

[46] Al-Aoukaty A., Appanna V.D., Huang J. (1991). Exocellular and intracellular accumulation of lead in *Pseudomonas fluorescens* ATCC 13525 is mediated by the phosphate content of the growth medium. FEMS Microbiol. Lett..

[47] Almagro V.M.L., Escribano M.P., Manso I., Saez L.P., Cabello P., Moreno-Viviána C., Roldán M.D. (2015). DNA microarray analysis of the cyanotroph *Pseudomonas pseudoalcaligenes* CECT5344 in response to nitrogen starvation, cyanide and a jewelry wastewater. J. Biotechnol..

[48] Cole M.A. (1979). Solubilization of heavy metal sulfides by heterotrophic soil bacteria. Soil Sci..

[49] Mokaddem H., Azouaou N., Kaci Y., Sadaoui Z. (2014). Study of lead adsorption from aqueous solutions on agar beads with EPS produced from *Paenibacillus polymyxa*. Chem. Eng. Trans..

[50] Murthy S., Bali G., Sarangi S.K. (2012). Biosorption of lead by Bacillus cereus isolated from industrial effluents. Br. Biotechnol. J..

[51] Saurav K., Kannabiran K. (2011). Biosorption of Cd (II) and Pb (II) ions by aqueous solutions of novel alkalophillic *Streptomyces* VITSVK5 spp. biomass. J. Ocean Univ. China (Oceanic Coast. Sea Res.).

[52] Cao Y., Zhang X., Deng J., Zhao Q., Xu H. (2012). Lead and cadmium-induced oxidative stress impacting mycelia growth of *Oudemansiella radicata* in liquid medium alleviated by microbial siderophores. World J. Microbiol. Biotechnol..

[53] Kalbe U., Berger W., Eckardt J., Christoph G., Simon F.G. (2007). Results of inter laboratory comparisons on the evaluation of the reproducibility of column percolation tests. J. Hazard. Mater..

[54] Kalbe U., Berger W., Simon F.G. (2009).

[55] Pang L., Close M.E., Noonan M.J., Flintoft M.J., Van den Brink P. (2005). A laboratory study of bacteria-facilitated cadmium transport in alluvial gravel aquifer media. J. Environ. Qual..

[56] Beveridge T.J., Murray R.G.E. (1980). Site of metal deposition in the cell wall of *Bacillus subtilis*. J. Bacteriol..

[57] Heipieper J., Cornelissen S., Pepi M., Timmis K.N. (2010). Handbook of Hydrocarbon and Lipid Microbiology.

[58] Torres S., Pandey A., Castro G. (2011). Organic solvent adaptation of Gram positive bacteria: applications and biotechnological potentials. Biotechnol. Adv..

[59] Rosenberg M. (2006). Microbial adhesion to hydrocarbons: twenty-five years of doing MATH. FEMS Microbiol. Lett..

[60] Marshall K.C. (1991).

[61] Panchanadikar V.V., Das R.P. (1994). Biosorption process for removing lead (II) ions from aqueous effluents using *Pseudomonas* sp. Int. J. Environ. Stud..

[62] Kobayashi H., Takami H., Hirayama H., Kobata K., Usami R., Horikoshi K. (1999). Outer membrane changes in a toluene-sensitive mutant of toluene tolerant *Pseudomonas putida* IH-2000. J. Bacteriol..

[63] Obuekwei C., Al-Jadi Z.K., Al-Saleh E. (2009). Hydrocarbon degradation in relation to cell-surface hydrophobicity among bacterial hydrocarbon degraders from petroleum-contaminated Kuwait desert environment. Int. Biodeterior. Biodegrad..

[64] Kłapcińska B. (1994). Binding of germanium and lead to *Pseudomonas putida* lipopolysaccharides. Can. J. Microbiol..

[65] Cabuk A., Akar T., Tunali S., Gedikli S. (2007). Biosorption of Pb(II) by industrial strain of *Saccharomyces cerevisiae* immobilized on the bio-matrix of cone biomass of *Pinus nigra*: equilibrium and mechanism analysis. Chem. Eng. J..

[66] Gabr R.M., Hassan S.H.A., Shoreit A.A.M. (2008). Biosorption of lead and nickel by living and non-living cells of *Pseudomonas aeruginosa* ASU 6a. Int. Biodeterior. Biodegrad..

[67] Salehizadeh H., Shojaosadati S.A. (2003). Removal of metal ions from aqueous solution by polysaccharide produced from *Bacillus firmus*. Water Res..

[68] Roane T.M. (1999). Lead resistance in two bacterial isolates from heavy metal contaminated soils. Microb. Ecol..

